# 4.H. Workshop: The Commercial determinants of health: latest thinking, models, tools and solutions

**DOI:** 10.1093/eurpub/ckac129.239

**Published:** 2022-10-25

## Abstract

*This abstract has been withdrawn*.

